# A new artificial intelligence system successfully detects and localises early neoplasia in Barrett's esophagus by using convolutional neural networks

**DOI:** 10.1002/ueg2.12233

**Published:** 2022-05-06

**Authors:** Mohamed Hussein, Juana González‐Bueno Puyal, David Lines, Vinay Sehgal, Daniel Toth, Omer F. Ahmad, Rawen Kader, Martin Everson, Gideon Lipman, Jacobo Ortiz Fernandez‐Sordo, Krish Ragunath, Jose Miguel Esteban, Raf Bisschops, Matthew Banks, Michael Haefner, Peter Mountney, Danail Stoyanov, Laurence B. Lovat, Rehan Haidry

**Affiliations:** ^1^ Division of Surgery and Interventional Sciences University College London London UK; ^2^ Wellcome/EPSRC Centre for Interventional and Surgical Sciences (WEISS) University College London London UK; ^3^ Department of Gastroenterology University College London Hospital London UK; ^4^ Odin Vision London UK; ^5^ NIHR Nottingham Digestive Diseases Biomedical Research Centre Nottingham UK; ^6^ Clínico San Carlos Madrid Spain; ^7^ UZ Leuven Leuven Belgium; ^8^ St Elisabeth Hospital Vienna Austria

**Keywords:** Barrett's Esophagus, artificial intelligence, convolutional neural networks, computer aided detection, neoplasia, AI, CAD, early neoplasia, early detection, CNN

## Abstract

**Background and aims:**

Seattle protocol biopsies for Barrett's Esophagus (BE) surveillance are labour intensive with low compliance. Dysplasia detection rates vary, leading to missed lesions. This can potentially be offset with computer aided detection. We have developed convolutional neural networks (CNNs) to identify areas of dysplasia and where to target biopsy.

**Methods:**

119 Videos were collected in high‐definition white light and optical chromoendoscopy with *i‐scan* (Pentax Hoya, Japan) imaging in patients with dysplastic and non‐dysplastic BE (NDBE). We trained an indirectly supervised CNN to classify images as dysplastic/non‐dysplastic using whole video annotations to minimise selection bias and maximise accuracy. The CNN was trained using 148,936 video frames (31 dysplastic patients, 31 NDBE, two normal esophagus), validated on 25,161 images from 11 patient videos and tested on 264 *iscan‐1* images from 28 dysplastic and 16 NDBE patients which included expert delineations. To localise targeted biopsies/delineations, a second directly supervised CNN was generated based on expert delineations of 94 dysplastic images from 30 patients. This was tested on 86 *i‐scan one* images from 28 dysplastic patients.

**Findings:**

The indirectly supervised CNN achieved a per image sensitivity in the test set of 91%, specificity 79%, area under receiver operator curve of 93% to detect dysplasia. Per‐lesion sensitivity was 100%. Mean assessment speed was 48 frames per second (fps). 97% of targeted biopsy predictions matched expert and histological assessment at 56 fps. The artificial intelligence system performed better than six endoscopists.

**Interpretation:**

Our CNNs classify and localise dysplastic Barrett's Esophagus potentially supporting endoscopists during surveillance.

## INTRODUCTION

Barrett's Esophagus (BE) is associated with increased progression risk to oesophageal cancer (EAC), progressing from non‐dysplastic Barrett's Esophagus (non‐dysplastic BE (NDBE)) to low grade dysplasia (LGD), high grade dysplasia (HGD) and then EAC. The overall 5‐year survival rate of EAC is less than 20%^1^ but early neoplasia in BE can be treated endoscopically with eradication rates of more than 90%.^2^

**Key summary**

**Summarise the established knowledge on this subject**

- Barrett's esophagus is associated with an increased risk of progression to esophageal cancer.
- Despite advances in endoscopic imaging early cancer in BE is still being missed.
- Studies have investigated neural networks for dysplasia detection using Fuji and Olympus systems. No studies have done so using Pentax imaging.

**What are the significant and/or new findings of this study?**

- Demonstrate a computer aided detection (CAD) system that is able to detect BE dysplasia with high accuracy on a per image and patient level and localise areas of interest with targeted biopsies with high sensitivities.
- This is the first study demonstrating an Artificial intelligence (AI) system in BE using the I‐scan/Pentax imaging system.
- We demonstrate and compare different approaches to development of algorithms for detection/localisation of BE to help identify the best approach to training these systems.

The current standard of care for BE surveillance is to take biopsies every 2 cm as part of the Seattle protocol. This is time consuming and may suffer from sampling error^3^ and poor compliance.^4^ Despite advances in endoscopic imaging, BE dysplasia is still missed.^5^ A meta‐analysis showed that amongst adults with NDBE at index endoscopy and prolonged follow up, 25% of EAC's are diagnosed within a year of the index procedure. They were classified as a missed diagnosis.^6^

There is growing interest in the use of computer aided detection (CAD) of early lesions in the gastrointestinal tract. To date, this has focused on detection of colonic polyps^7^, ^8^, ^9^ but CAD is likely to become increasingly important for BE neoplasia detection and several studies in recent years investigate this.^10^, ^11^ Artificial intelligence (AI) technology could lead to less random biopsies with lower histopathology costs, shorter procedures and early endoscopic therapy could offset the costs of an oesophagectomy/radiotherapy. This is increasingly important in view of the COVID‐19 pandemic induced reduction in routine endoscopic provision.^12^ There may still be an issue with the detection of invisible dysplasia. This will be answered with randomised control trials where seattle protocol biopsies are compared against AI predictions.

With advances in endoscopic optical technology, classification protocols have been developed based on vascular architecture and mucosal pit pattern to improve dysplasia detection.^13^, ^14^, ^15^ It might therefore be possible to train a CAD system to use these features.

*I‐scan* (Pentax Hoya, Japan) is a virtual chromoendoscopy technique that uses post processing technology to provide contrast and surface enhancement. There are 3 modes – *i‐scan 1* (surface enhancement), *i‐scan*‐2 (contrast enhancement), *i‐scan* 3 (tone enhancement). *I‐scan 1* has become the default equivalent of high‐definition white light (WL) with the Pentax system. It is the best imaging mode for lesion detection (Supplementary Figure 1).^16^ Previous publications from our group have shown that utilisation of optical enhancement with *i‐scan* is superior to WL in BE and therefore has paved the way for *i‐scan* to be the standard of care in most units using this platform.^15^, ^17^

**FIGURE 1:**
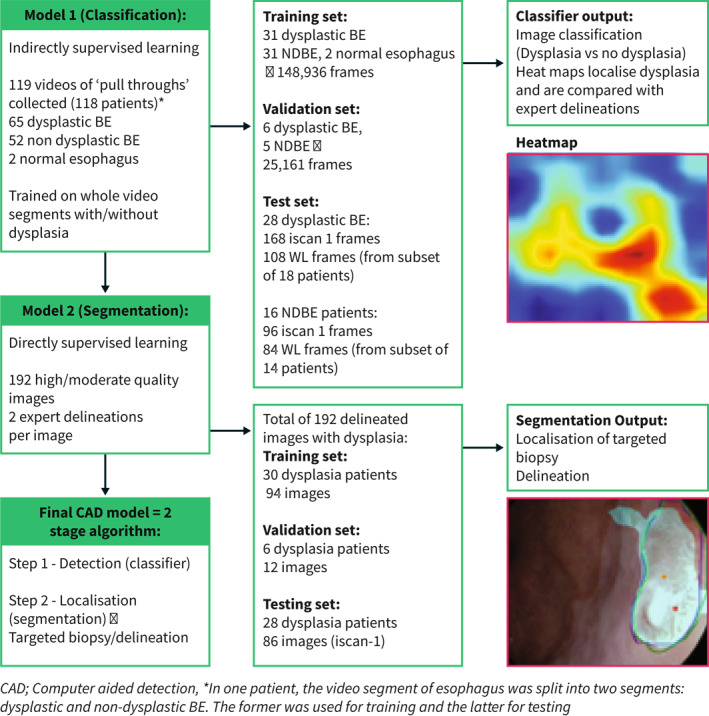
Breakdown of the data set in the classification/segmentation models and the potential importance of each model output in the computer aided detection (CAD) system. CAD; Computer aided detection, *In one patient, the video segment of esophagus was split into two segments: dysplastic and NDBE. The former was used for training and the latter for testing

Studies have investigated development of neural networks for dysplasia detection in the Esophagus with some promising results using Olympus and Fuji imaging.^10^, ^18^ However, to the best of our knowledge, no studies have developed a neural network using Pentax *i‐scan* imaging. The studies to date have trained neural networks on limited number of high quality still images. These lack generalisability, particularly as most of the testing and training data sets originated from the same centre or country.^19^, ^20^ These factors may limit real time implementation.^21^

The primary aims of this multi‐centre study were first, to develop a neural network to detect dysplasia within BE by classifying an image as dysplastic or non‐dysplastic and second to identify a point of interest for targeted biopsies. Secondary aims were to achieve these goals fast enough to allow real‐time dysplasia detection, compare the performance of the system on i‐scan 1 versus WL images and compare the performance versus endoscopists.

## METHODS

### Patient recruitment

Patients attending for BE assessment at four expert European centres were recruited. All cases were collected prospectively including 86 that were collected prospectively in a previous BE imaging study.^15^, ^17^, ^22^ Patients were excluded if there was evidence of oesophageal varices, strictures or oesophageal ulceration. The study was approved by the Cambridge central research ethics committee (REC Reference No. 18/EE/0148) for UK sites. European centres received ethical approval from local committees for use of images for this and other imaging‐based research projects.

### Endoscopic procedures and video collection

All videos and still images were collected by six expert endoscopists (RJH, MRB, VS, RB, JM, KR). We defined these as endoscopists with more than 5 years' experience of BE endotherapy and who perform endotherapy procedures weekly in BE expert centres as defined by the European Society of Gastrointestinal Endoscopy guidelines.^23^

Videos were prospectively collected using the Pentax endoscopy system (OPTIVISTA plus, EPK‐i7000, EG‐2990i, EG29‐i10). Mucus lining the Esophagus was removed using a simeticone and water solution. The endoscopist then performed a ‘pull through’ withdrawal of the endoscope from the gastroesophageal junction to the maximal extent of BE. Procedures were recorded in high‐definition WL and *i‐scan* one imaging modes in patients with dysplastic lesions in BE (LGD/HGD/EAC) and patients with NDBE. *I‐scan 1* was the default imaging modality in the collected data in all centres. ‘Pull throughs’ were also collected in WL for most patients.

### Tissue acquisition and histology

BE with no suspicion for dysplasia was biopsied as per the Seattle protocol. Areas suspicious for dysplasia either were target biopsied or resected by endoscopic mucosal resection (EMR). Histology was reviewed by expert BE histopathologists with more than 10 years' experience. Cases of suspected dysplasia were reviewed by two different BE histopathologists in each expert centre.

### Creation of a gold standard on video segments

A computer vision annotation tool (Odin Vision, London, UK) was used to annotate a sequence of video frames. Annotation confirmed that dysplasia was present within individual frames without defining the position. Gold standard was determined from histology of EMR specimens or biopsies and the annotation segments matched these areas on the videos. In NDBE patients, all the frames from the Esophagus, including squamous mucosa, were included. The dysplastic and non‐dysplastic frames were used to train and validate a classification convolutional neural network (CNN; Supplementary Figure 2).

**FIGURE 2:**
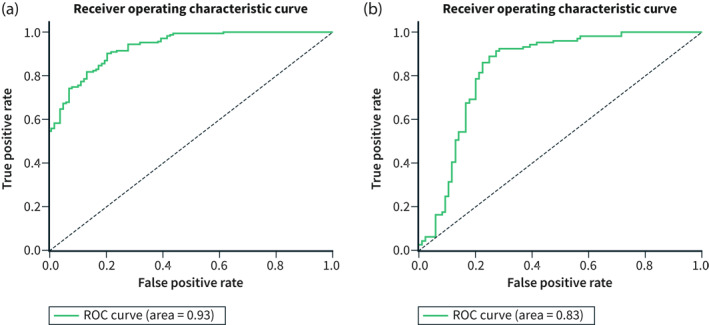
AUC performance of the classifier algorithm on iscan‐1 (a) and unenhanced white light (WL) (b)

### Creation of a gold standard on still images

High and moderate quality BE images were delineated for the presence of dysplasia by three expert Barrett's endoscopists (RH, MB, VS; Supplementary Figure 2). High quality images were defined as images with clear views of lesions within a distended Esophagus and no artefact (blood/mucus). Moderate quality images contained some artefacts but there remained reasonable views of the lesion to make a diagnosis. The quality of images was determined by the same clinician on the study team (MH).

Each image was delineated by two of the three expert endoscopists. The delineated areas all had confirmed dysplasia on histology. Delineations were used to test the performance of the CNN's. We test the performance of the targeted biopsy predictions generated by the CNN on two levels ‐ against all areas annotated by experts and then against the small area of overlap between experts.

### Model 1: Classification convolutional neural network for dysplasia detection within BE

We trained a CNN with a Resnet101 architecture to classify images into dysplastic or non‐dysplastic using randomly selected frames from annotated videos. For each pixel, the CNN predicted a number between 0 (no dysplasia) and 1 (dysplasia present). Further algorithm development details can be found in the supplementary section.

A directly supervised learning classification network is trained on BE images which have been individually delineated for areas of dysplasia (methodology for training model 2). We derived heat maps for areas of interest from the classification CNN using an indirectly supervised learning approach. This relies on informing the model whether an image contains dysplasia (Methodology for training model 1). The network works out what constitutes an abnormality for itself.

#### Classification model data set for dysplasia detection within BE

One hundred eighteen different patients were included. Lesions were randomly split (computer generated) into training, validation and testing sets with no overlap of data. Each set was stratified to ensure consistent proportions of patients (Figure 1). The network was trained using a total of 148,936 frames. No areas of dysplasia were specifically delineated. A heatmap was generated which showed the likelihood of dysplasia in each pixel in the image. In the testing set of 44 patients, six *i‐scan one* images and six WL images were randomly selected per patient. Two expert delineations per image on 86 dysplastic images from 28 patients helped test the reliability of the computer‐generated heat map outputs. Refer to supplementary Table 1 for a breakdown of the test set based on location, histology and Paris classification.

**TABLE 1:** Performance metrics of the classifier model on iscan‐1 and unenhanced white light (WL) imaging in the test data set

Tested on	AUC	Sens	Spec	Accuracy	Number of dysplastic patients	Number of NDBE patients	No. of dysplastic images	No. of NDBE images
*I‐scan 1*	93%	91%	79%	86%	28	16	168	96
Unenhanced WL	83%	92%	73%	83%	18	14	108	84

*Abbreviations*: AUC; area under the receiver operator curve, WL; White light, Sens; Sensitivity, Spec; Specificity.

### Model 2: Segmentation convolutional neural network for localisation of dysplasia within BE with targeted biopsies and delineations

We trained a model with a FCNResnet50 architecture to classify pixels as dysplastic or non‐dysplastic using still images with an expert delineation of an area of dysplasia. The backbone of the model was trained from an external data set (Gastrointestinal Artificial Intelligence Diagnostic System).^24^ The output was a segmentation map where pixel values ranged between 0 (no dysplasia) and 1 (dysplasia present). The gold standard was two expert delineations per still image which matched with areas of histologically confirmed dysplasia (Supplementary Figure 2). Further algorithm details can be found in the supplementary section.

#### Segmentation model data set for localisation of dysplasia within BE with targeted biopsies and delineations

One hundred ninety‐two images containing BE dysplasia from 64 different patients were each delineated by two experts for dysplastic areas. These images were randomly selected. Further model details are in Figure 1. The patients in the independent training, validation and testing set for this model were allocated to the same three groups in the classification model to minimise bias with no overlap of data or patients. The delineations of one expert were used to train the model. In the testing set the delineations of all experts were used to test the output predictions of the CNN versus the union of expert delineations and area of overlap. For each image of BE dysplasia, the model generated a prediction for delineation of the area of dysplasia and targeted biopsies.

### Comparison of the performance of the convolutional neural network versus endoscopists

Sixty one i‐scan one images from the testing set were randomly selected (28 dysplastic, 33 non dysplastic). Six non expert endoscopists with greater than 3 years of endoscopic experience assessed for the presence/absence of dysplasia on each image. The performance of the CNN in detecting dysplasia was compared to endoscopists.

### Statistical analysis

Descriptive statistics consisted of the mean (+/− standard deviation). We measured the sensitivity and specificity at a per‐image and per patient level. The area under the receiver operator curve (AUC) was calculated. Further details are in the supplementary section.

## RESULTS

### Model 1: Classifier

#### Per image classification

In the test set the neural network detected dysplasia on *i‐scan 1* images with an AUC of 93%, sensitivity of 91% and specificity of 79%. The AUC was 10% greater than on WL (Table 1; Figure 2). The i‐scan 1 and WL experiments were on the same patients but were two independent experiments where the training data set of i‐scan 1 was larger.

**FIGURE 3:**
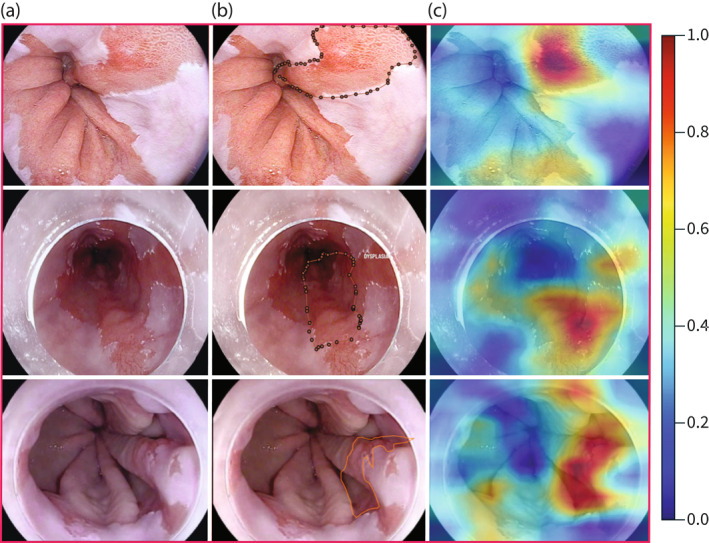
Heat map outputs from the classifier model trained on video frame segments without delineations using an indirectly supervised approach. (a) Original image, (b) expert delineation, (c) heat map generated by the classifier. On the heat maps, the pixels are coloured based on their dysplastic content according to the model. Red areas (closer to 1), show the most likely dysplastic pixels and therefore optimal area for a targeted biopsy

Heat maps generated from the classifier trained on video segments, once thresholded, overlapped with at least one expert delineation in 98% of the *i‐scan 1* test set images where a true diagnosis of dysplasia was made based on a minimum of 1 pixel of overlap (Figure 3). With a Dice coefficient overlap greater than 20%, 78% of the heat maps overlapped with the union of experts at this threshold.

#### False negative and positive classification results on i‐scan 1 images

Sixteen out of 168 dysplastic *i‐scan one* images in the testing set had a false negative classification, and 11/16 of these images were from three patients. Twenty out of 96 NDBE *i‐scan one* images showed a false positive classification, and 18/20 of the false positives were from three patients (Supplementary Figure 3).

#### Classification based on histology on i‐scan one images

In the test data set 15 patients had EAC, 11 had HGD, two patients had LGD and 16 patients had no dysplasia on histology. The CNN achieved a per image sensitivity of 91% which was almost identical in all the subgroups (90% EAC; 91% HGD; 92% LGD) and a per image specificity of 79% in the 16 patients with no dysplasia.

#### Per patient classification results

Using a minority voting approach on *i‐scan 1* images, where at least 2 of the 6 images presented for each patient were correctly diagnosed as dysplastic, the CNN achieved a per patient sensitivity of 100%. Using a majority voting approach where at least 4 of 6 images were correctly diagnosed, the sensitivity for dysplasia detection was 89.3%.

### Model 2: Localisation of dysplasia in BE

#### Localisation of points of interest for targeted biopsies

Four different scenarios for localisation of points of interests were generated (Table 2). A targeted biopsy was deemed correct if it fell within the area outlined by expert delineation which matched histology. Further information of algorithm development is in the supplementary section.

**TABLE 2:** Targeted biopsy predictions generated by the CNN

	Mean number of biopsy predictions by the CNN	Maximum number of biopsy predictions by the CNN	Proportion of biopsies within expert 1 delineation	Proportion of biopsies within expert 2 delineation	Proportion of biopsies within intersection of delineations	Proportion of biopsies within union of delineations
Scenario 1 (1 max pix value)	1	1	81%	74%	71%	85%
Scenario 2 (1 max pix value + 1 geometric centre)	2	2	88%	81%	78%	91%
Scenario 3 (1 max pix value + up to 2 geometric centres)	3	3	91%	85%	81%	93%
Scenario 4 (1 max pix value + all geometric centres)	3	5	94%	90%	86%	97%

*Note*: Different scenarios were generated assessing where the targeted biopsies fall within the gold standard expert delineations which matched areas of histologically confirmed dysplasia in videos. Based on expert consensus scenario 2 was considered the most clinically relevant and user friendly at the same time.

**FIGURE 4:**
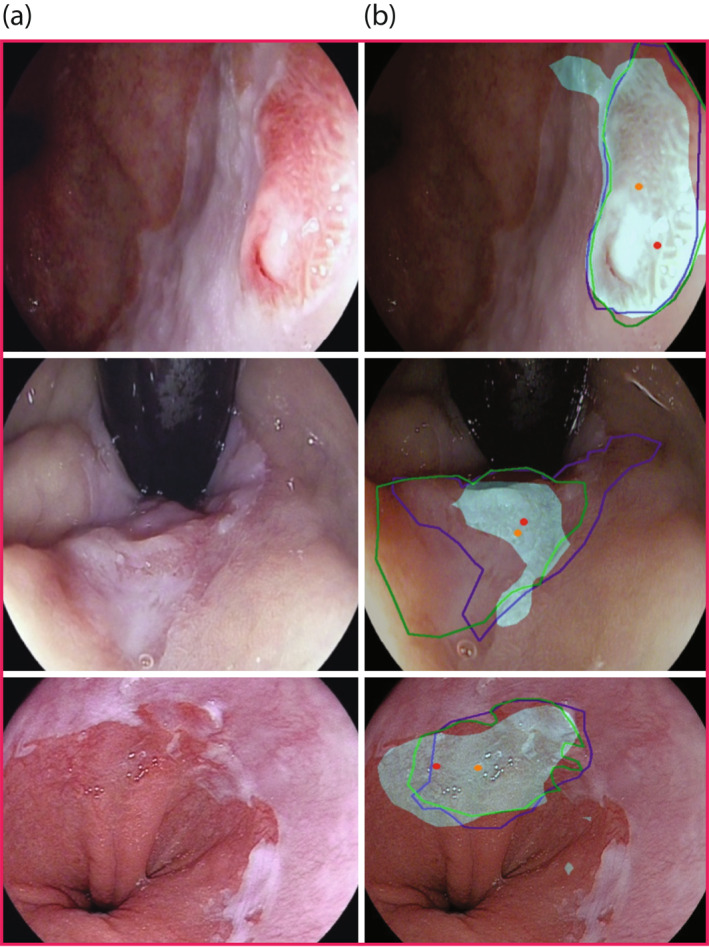
Images with BE dysplasia (a) and targeted biopsy and delineation predictions relative to the expert ground truth (b) by the Artificial intelligence (AI) system. Delineations (Green and purple outline) = 2 different expert delineations. Blue shaded delineation = delineation prediction by the convolutional neural network (CNN). Orange and red dot = point of interest/targeted biopsy predicitons by the AI system based on scenario 2 (Table 2)

Expert consensus determined that the optimal scenario would be to recommend two points of interests for biopsies. This balances the number of necessary biopsies with high performance. Based on this, the system recommended targeted biopsies in areas of the union of expert delineations with a sensitivity of 91% in the model two test set of 28 dysplastic patients and 78% in the intersection of expert delineations (Figure 4, Table 2). When allowing the system to generate any number of targeted biopsy predictions, the sensitivity for localising dysplasia was 97% in the union of expert delineations (Table 2).

#### Localisation with delineation

The delineation outline prediction for an area of dysplasia generated by the segmentation model overlapped with at least one expert delineation in 98% of images with a minimum of one pixel of overlap (Figure 4b). This suggested the model is pointing in the right direction. The segmentation delineation had a 50% average Dice score with expert 1 (including the false negative predictions in this result).

### Speed of detection of BE dysplasia

The mean (+/− standard deviation) time for the classifier analysis of each image was 0.021 s per image (+/− 0.008), or 48 frames per second (fps). The mean time for the segmentation network to delineate dysplasia on each image was 0.018 s (+/− 0.006) or 56 fps.

### Comparison of the performance of the convolutional neural network versus endoscopists in the detection of dysplasia on i‐scan 1 images

On a subset of testing set images 6 non expert endoscopists detected dysplasia with a mean sensitivity of 79% and specificity of 49%. The CNN classified dysplasia with a sensitivity of 96% and specificity of 88% on the same images (Table 3).

**TABLE 3:** Comparison of the performance of the Artificial intelligence (AI) system versus 6 non expert endoscopists

	Per image sensitivity	Per image specificity	Mean
Endoscopist 1	23/28 (82%)	19/33 (58%)	
Endoscopist 2	21/28 (75%)	12/33 (36%)	Sensitivity = 79%
Endoscopist 3	16/28 (57%)	22/33 (67%)	
Endoscopist 4	24/28 (86%)	22/33 (67%)	Specificity = 49%
Endoscopist 5	22/28 (79%)	15/33 (46%)	
Endoscopist 6	26/28 (93%)	7/33 (21%)	
AI system	27/28 (96%)	29/33 (88%)	

## DISCUSSION

We demonstrate for the first time a CAD system which can accurately detect early neoplasia in BE using Pentax endoscopes. It has a per image sensitivity and specificity of 91% and 79%, respectively, on *i‐scan 1* imaging. The heat map generated by the indirectly supervised learning algorithm, once thresholded, overlaps with at least one expert endoscopist in 98% of images with a minimum of one pixel of overlap. We have developed a second, directly supervised, CNN which can localise points of interest to detect dysplasia with a sensitivity of up to 97%.

In current practice endoscopists perform ‘pull throughs’ in the esophagus during BE surveillance and take targeted biopsies. An indirectly supervised machine learning approach, can potentially be a promising methodology to identify an area of interest in BE with heat map overlays. It works fast, within 20 milliseconds, without the need for specialised expert delineations of every frame as in previous studies.^19^ This however needs a much larger data set and further studies to assess its true value in terms of localisation performance. We developed a second segmentation algorithm which allows a high accuracy for localising targeted biopsies with high detection speeds. This creates a two‐stage CAD algorithm which can be translated into a working unit in real time where the system generates predictions on freeze frames taken as per standard of care (Supplementary Figure 4). We use two models because of annotated data availiability. To use a delineation model to both, detect and delineate more data is needed. In terms of real‐time analysis, delineation models are slower than classification ones. Our current set up allows for faster, real time, classification than with a single delineation model, and a slower (but still fast) delineation on demand.

We chose to use an indirect learning approach to generate heat map outputs from the classifier to assess whether they would help endoscopists to identify areas of interest in BE without the need of expert delineations to train, to provide insight into the classifier's predictions, and to compare to the segmentation output using a directly supervised learning approach to assess the potential implications on the way we train and process data when developing BE CAD systems. The outputs suggest that the model focuses on important dysplastic features.

Van der Sommen et al developed a computer algorithm using 100 still images. The system identified early BE dysplasia with a sensitivity and specificity of 83%.^19^ This is much earlier work and further work has been done more recently. Von Ebigbo et al.^20^ demonstrated a promising CNN trained on still images which detects early BE cancer. De Groof et al produced a promising CAD system using five independent endoscopy sets with high quality BE images.^11^ We trained the classification neural network on a large number of images (148,936 frames) from a large number of individual patients (*n* = 64) each with a video assessment. We also trained on still images like in previous studies but use multiple frames per video to maximise the training data set and provide more valuable information for the CNN's. The CNN achieved high sensitivities in detecting EAC and HGD but also LGD. Previous studies did not include LGD. We felt it was important to include this subgroup in our data as these lesions are often upstaged on EMR to HGD or EAC.

Previous studies trained and tested networks on Olympus and Fujinon endoscopy systems for the detection of BE.^10^, ^11^, ^18^ Ours is the first study developing a CNN using the Pentax system. *I‐scan one* is often the default imaging modality on these systems as this provides the natural colour tone of WL and added advantage of surface enhancement. Therefore, the training set was predominantly composed of *I‐scan 1* as opposed to WL. Our results hint towards improved performance of the classifier model on *i‐scan 1* versus WL. However, of note the data set for i‐scan 1 was larger than that of WL and matched studies are needed to directly compare the performance of the two.

Seattle protocol biopsies on a surveillance endoscopy will sample a small surface area of a BE segment.^6^ We developed a CNN using a segmentation algorithm which is able to localise dysplasia with point of interest predictions with an accuracy of up to 97%. Biopsy positive patients can be referred for curative endotherapy. An AI system for BE is more useful in district general hospitals where the targeted biopsy function during a pull through will be useful. Experts in teaching centres will then perform a resection relying more on magnification imaging to delineate lesions.

Studies have developed systems which are able to detect dysplasia in BE at varying speeds. De Groof et al.^10^, ^11^ developed systems which were able to detect dysplasia at a speed of 1.051 and 0.191 s per image. Our network performed in real time with a mean speed of 0.021 s per image. To fairly compare the speed of the systems, they need to be benchmarked on the same machine. However, the results show that our system will be able to support endoscopists' decision‐making in real time.

Six images per case were selected for the testing set to simulate the clinical workflow, where relevant endoscopic images would be taken. A previous pilot study tested the performance of a CAD system during live endoscopy assessment of BE on freeze frames. They used a majority and minority voting approach to label an area of BE as dysplastic.^25^ The per patient sensitivity was 90% in both analyses. In our current model using a minority and majority voting approach where at least 2/6 and 4/6 correctly classified images are necessary, we achieve a per patient sensitivity of 100% and 89.3% respectively on *i‐scan one* images.

The CAD system in our study performed better than all 6 non expert endoscopists with a range of 3–11 years of endoscopic experience. This is the cohort for whom a BE CAD system would be most beneficial. A smaller subset of images was randomly selected for this experiment as it will mean endoscopists will be more likely to complete the task. The AI system performed better on this subset however when comparing the endoscopists performance to the AI system performance on the whole data set it is still weaker.

There are limitations in this current study. We developed a model which is trained using videos from a single endoscopic system. Ideally, to allow for more generalisation, we would incorporate data from other systems. The current CNN is tested on higher quality still images. However, our training strategy and data selection would allow us now to develop networks which could potentially work better on whole video data in real time. Another limitation is the threshold for neoplasia detection was based on the test set. However, the performace can be compared independently of the threshold selection by looking at the AUC scores. It is important to test the threshold selection on an independent, hold out, data set, which was not available at the time. Another limitation is the testing set in ‘model 2’ (segmentation) was 86 images. Ideally, we would have selected a larger test set however in this model these images were all required to be delineated by experts. For the purposes of the available time of experts and to complete the task a smaller data set of images was selected. This problem could be rectified in future by selecting a broader range of experts. The data set for i‐scan 1 was larger than that of WL. This should be kept in mind when looking at the improved performance on i‐scan 1 versus WL in the classification of dysplasia. In future we will perform matched studies comparing the two light modalities where both cohorts are matched in terms of number of images, histology and Paris classification of lesions. Another potential limitation is the segmentation model was trained based on the delineations of one expert. In future to help improve our results further we will aim to train the model using the intersection of expert delineations to improve the localisation ability of the CAD system.

We demonstrate a CAD system which is able to detect BE dysplasia with high accuracy on a per image and per patient level. It localises areas of interest with targeted biopsies with high sensitivities. Our next step is allowing this to work in real time with whole video predictions.

## CONFLICT OF INTEREST

MH: Speaker fees (Cook Medical). JGP,DL, DT and PM: Employees at Odin Vision. DS: Share holder Odin vision and Digital Surgery Ltd. LBL: Consultancy and minor share holder Odin Vision. RH: Educational grants to support research infrastructure from Medtronic ltd. Cook endoscopy (fellowship support), Pentax Europe, C2 therapeutics, Beamline diagnostic, Fractyl Ltd.

## Supporting information

Supplementary Material

## Data Availability

The data that support the findings of this study are available from the corresponding author upon reasonable request.
