# Supplementary material for: A new artificial intelligence system successfully detects and localises early neoplasia in Barrett's esophagus by using convolutional neural networks
Source: United European Gastroenterol J. 2022 May 6;10(6):528–37. doi: 10.1002/ueg2.12233 (PMC9278593; doi:10.1002/ueg2.12233)
Supplement: Supplementary file 1 — Supplementary Material [file UEG2-10-528-s001.docx]

**Supplementary file**

**Section 1**

**Model 1: Classification convolutional neural network for dysplasia detection within BE (Classification algorithm development (Resnet101))**

Data pre-processing and augmentation techniques

During training, random sampling of 5000 frames was performed on each epoch to minimise overfitting, re-sampling at every new epoch. The augmentation operations consisted of random affine transformations (rotation, translation and scale) and random colour transformations (brightness, contrast, saturation and hue) to avoid overfitting. Finally, the images were pre-processed by cropping out the video borders, followed by resizing the images to 512 by 512 pixels, and an intensity normalization step using the ImageNet channel values.

Hyperparameters and training

The model was trained initialising the backbone weights from ImageNet. It was trained for a maximum of 100 epochs, selecting the model with the highest accuracy in the validation set. For training, a balanced dataset was created with equal numbers of dysplastic and non-dysplastic frames from videos.

Training parameters:

Optimizer: Stochastic Gradient Descent, learning rate: 0.0001, batch size: 14, Image input size: 512x512px, loss: cross-entropy

Post-processing

The model was trained to classify over two classes: dysplastic and non-dysplastic. The output from the dysplastic class was thresholded to obtain a final prediction. The threshold was selected on each testing (different lighting modalities – iscan-1 and white light) set as to obtain a minimum sensitivity of 90%. We used the same threshold in different centres. The processing speed of the model was measured on a GeForce RTX 2080 Ti (11GB) Graphics processing unit (GPU). The methods used to generate the heatmaps was gradcam ^1^. The obtained heatmaps were generated by using the gradients corresponding to the dysplastic class.

**Section 2**

**Model 2: Segmentation convolutional neural network for localisation of dysplasia within BE with targeted biopsies and delineations (Segmentation algorithm development (FCNResnet50))**

Data pre-processing and augmentation techniques

During training, the augmentation operations consisted of random affine transformations (rotation, translation and scale) and random colour transformations (brightness, contrast and saturation). Finally, the images were pre-processed by cropping out the video borders, followed by resizing the images to 512 by 512 pixels, and an intensity normalization step using the ImageNet channel values.

Hyperparameters and training

During training, an additional auxiliary segmentation head received features from the third backbone building block^2^. These feature maps had undergone fewer pooling steps, and therefore were twice the size of the main backbone’s feature maps. Thus, the corresponding segmentation output before the interpolation layer was double the resolution of the main segmentation output. A combination L aux + L main of the losses computed from the two segmentation heads was used as the final loss L, allowing to refine the spatial precision of the output. The model was trained initialising the backbone weights from a task to classify images from an external dataset^3^ as cancerous or non-cancerous. The rest of the model weights (the segmentation head) were initialised randomly. It was trained for a maximum of 1000 epochs, selecting the model with the highest pixel accuracy from the tuning set.

Training parameters

Optimizer: Stochastic Gradient Descent, learning rate: 0.001, batch size: 8, Image input size: 512x512px, loss: cross-entropy

Post-processing

The model was trained to output a segmentation map for each input frame where pixel values ranged between 0 (no dysplasia) and 1 (dysplasia present). The processing speed of the model was measured on a GeForce RTX 2080 Ti (11GB) GPU.

**Scenarios for model development for targeted biopsy predictions**

Four different scenarios for localisation of points of interests were generated using: (i) the maximum positive pixel value in the raw predicted segmentation output, and (ii) the geometric centre of the segmentation delineation which can be one or multiple points depending on the predicted segmentation.

Each image contains the following information **(Figure 4):**

1. Two expert delineation outlines of where the clinicians think there is dysplastic barrett's tissue (blue and green outlines) which matches with histology gold standard
2. The thresholded (0.5) heatmap segmentation output from the model (light blue shaded area)
3. Points of interest dots

**Section 3**

**Statistical analysis**

We defined a prediction for the presence of dysplasia at a patient level using a minority and majority voting approach. The minority voting approach called the patient positive if dysplasia was correctly predicted in at least 2/6 images for that patient. The majority voting approach required correct prediction of dysplasia in at least 4/6 images in the same patient. We also calculated the Sorensen-Dice coefficient. We used it to determine the degree of overlap between the heatmaps generated by the classifier model and the expert gold standard delineations.

The Dice score is a traditional way of computing overlap between delineations from AI systems, computed as:

Dice = (2*TP)/ (2*TP+FP+FN)

where TP, FP, and FN are the total number of true positive, false positive and false negative pixels in an image respectively. A true positive pixel is a pixel where both the expert and the AI detected dysplasia, a false positive is where only the AI detected dysplasia and a false negative is where only the expert detected dysplasia.

**Supplementary tables**

**Supplementary table 1: Breakdown of the classifier testing data set based on location, histology and Paris classification of any lesions.**

|  | Location | | | Histopathology | | | | Paris classification of dysplastic lesions | | |
| --- | --- | --- | --- | --- | --- | --- | --- | --- | --- | --- |
|  | UK | Belgium | Spain | IMC | HGD | LGD | NDBE | Paris 2a | Paris 2b | Paris 2c |
| Number of patients | 36 | 3 | 5 | 15 | 11 | 2 | 16 | 16 | 9 | 3 |

UK = United Kingdom, IMC = intramucosal cancer , HGD = high grade dysplasia, LGD = low grade dysplasia, NDBE = non dysplastic Barrett’s esophagus

**Supplementary Figures:**

**Supplementary Figure 1: I scan 1 improves detection of lesion borders and mucosal texture. An area of HGD in BE at three o’clock can be delineated more clearly on *i-scan 1* (B) compared to unenhanced white light (A).**


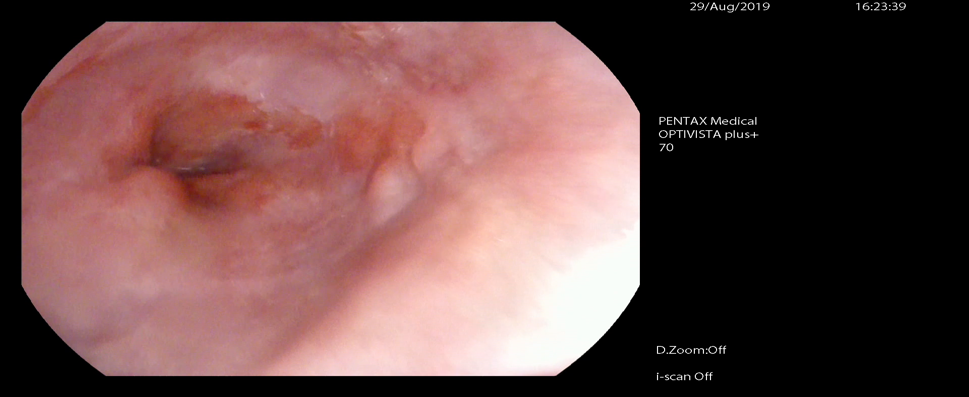

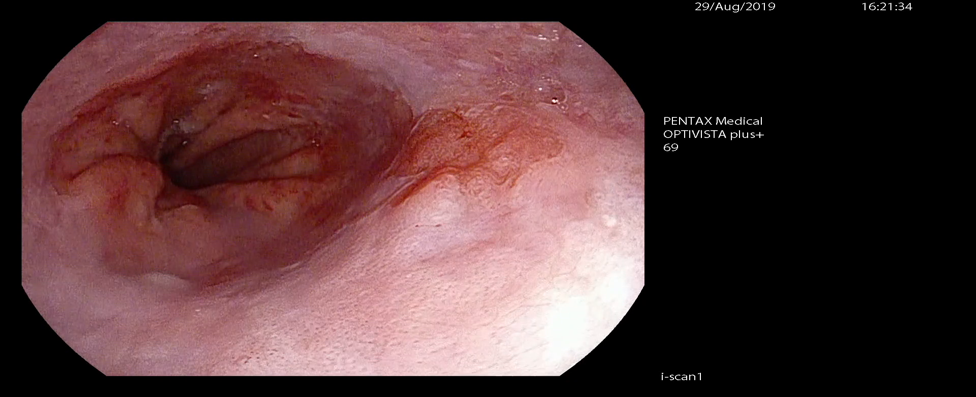


**Unenhanced White light (A) *I-scan 1* (B)**

**
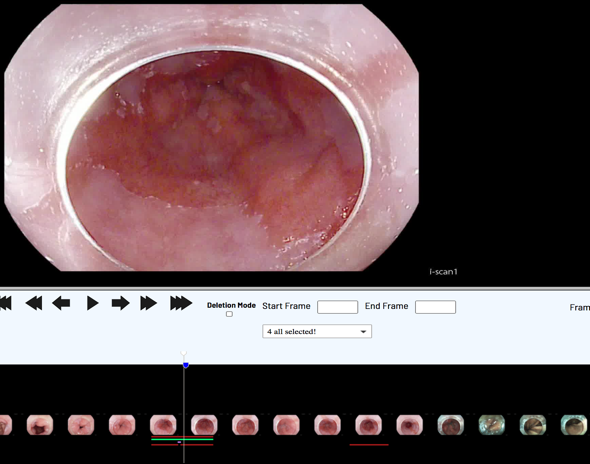

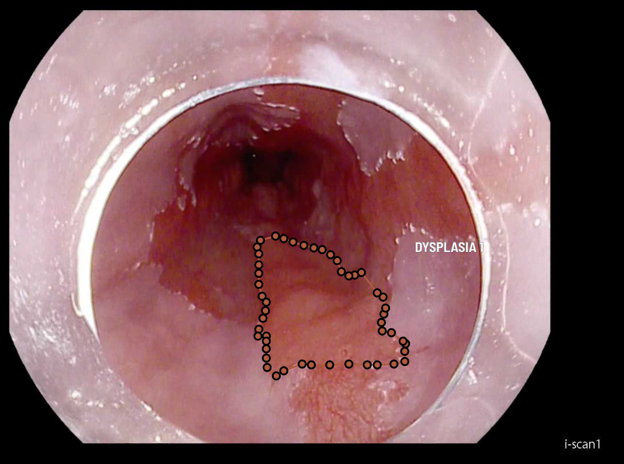
Supplementary Figure 2: (A) Annotation of segments of videos. Blurry images were included to reflect real word data. (B) Expert delineation of dysplasia/early cancer in the Esophagus which matched histology.**

**
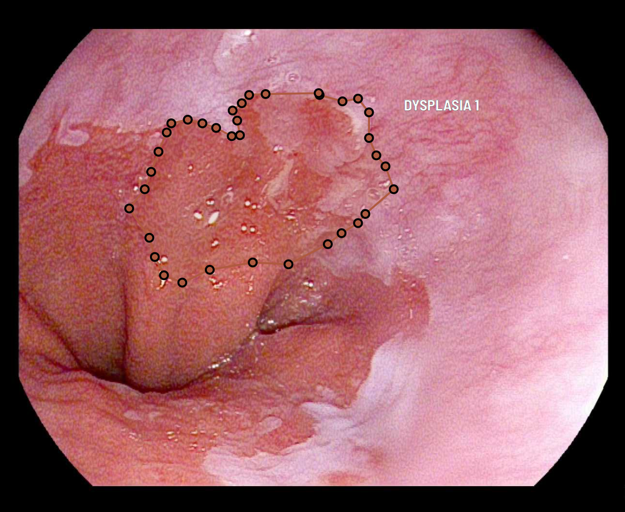

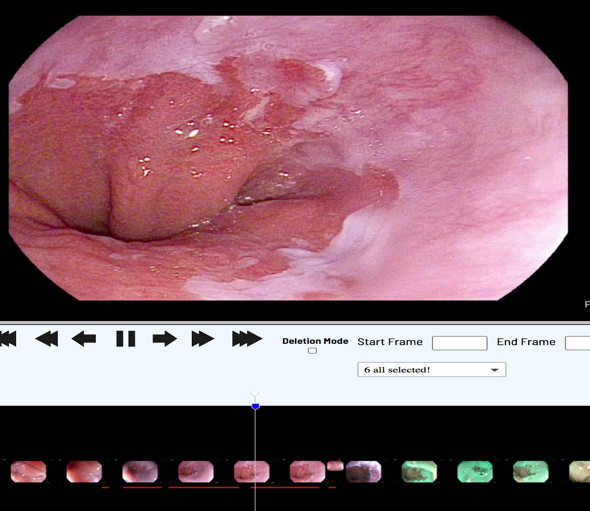
**

**A B**


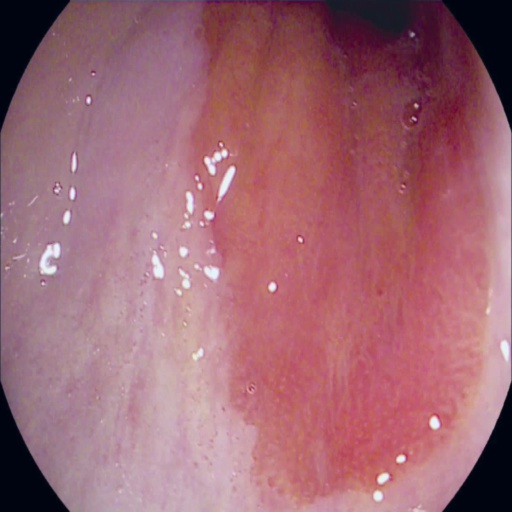

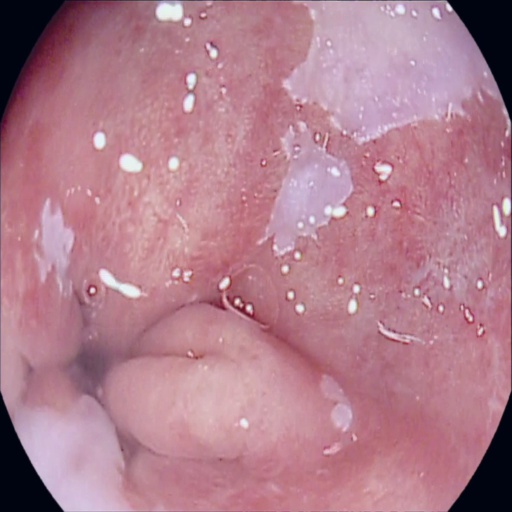
**Supplementary Figure 3: Example of a false positive classification prediction (A) due to a prominent gastric fold at the gastroesophageal junction. (B) a false negative prediction in an area of subtle, flat high-grade dysplasia.**

**A B**

**Supplementary figure 4: Proposed two-stage algorithm for the detection and localisation of dysplasia in BE**


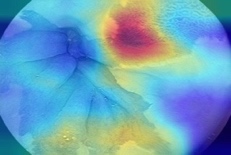


**Classifier**:

Take freeze frames during a pull through. Is there dysplasia in the esophagus?

No

Yes

**Segmentation**:

- Where in the esophagus is it?
- Target biopsy or delineate area of interest

Continue endoscopic assessment of BE


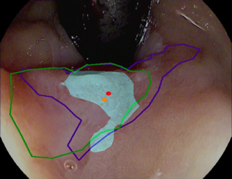


Support endoscopists decision making: Target biopsy an area of interest. If positive for dysplasia a referral can then be made for endoscopic resection. Experts will use the support of magnification images to delineate the edges of lesions for endoscopic resection.

**References**

1. Selvaraju R, Cogswell M, Das A et al. Grad-cam: Visual explanations from deep networks via gradient-based localization. Proceedings of the IEEE international conferences on computer vision 2017
2. Evan S, Long J, Darrell T. Fully convolutional networks for semantic segmentation. Proceedings of the IEEE conference on computer vision and pattern recognition 2015
3. Luo H, Xu G, Li C et al. Real-time artificial intelligence for detection of upper gastrointestinal cancer by endoscopy: a multicentre, case-control, diagnostic study. Lancet Oncol 2019; 12 (20): 1645-54.
